# Meningitis retention syndrome

**DOI:** 10.3402/jchimp.v2i1.15761

**Published:** 2012-04-30

**Authors:** Abhishek Krishna, Pavan Devulapally, Ibrahim Ghobrial

**Affiliations:** Department of Internal Medicine, University of Pittsburgh Medical Centre-McKeesport Hospital, McKeesport, PA, USA

**Keywords:** aseptic meningitis, atonic bladder, Elsberg syndrome, herpes simplex 2 virus infection, acyclovir, polyradiculitis, radiculomyelitis

## Abstract

A 50-year-old Caucasian woman presented with signs and symptoms of meningitis preceded by a 3 day history of flu-like symptoms and progressive difficulty with urination. Cerebrospinal Fluid (CSF) analysis was consistent with aseptic meningitis. She was found to have a significant urinary retention secondary to atonic bladder. MRI of the brain and spine were normal and CSF-PCR (polymerase chain reaction) was positive for HSV-2. Urinary retention in the context of meningitis and CSF pleocytosis is known as Meningitis Retention Syndrome (MRS). MRS is a rare but important complication of meningitis most commonly associated with HSV-2. Involvement of central pathways may have a role in the pathogenesis of MRS but this is poorly documented. MRS is different from Elsberg syndrome wherein patients display features of lumbosacral polyradiculitis or radiculomyelitis. Early treatment with antiviral therapy was associated with a favorable outcome in our patient.

The development of urinary retention in the context of meningitis and CSF pleocytosis without any lumbosacral radiculomyelitis is known as Meningitis Retention Syndrome (MRS) (1).

On the other hand, the combination of acute urinary retention, herpetic genital vesicles, lumbosacral radicular pain, hypoesthesia and muscle weakness, was described over 75 years ago and is referred to as Elsberg Syndrome (2).

Both conditions are very rare and have been reported in association with Herpes Simplex-2 (HSV2). MRS has also been reported to be caused by other organisms like mycobacteria and listeria.

## Case report

A 50-year-old Caucasian woman presented with severe throbbing headache, neck stiffness and photophobia for 1 day. She also had a 3 day history of flu-like symptoms as well as progressive difficulty with urination. On examination, she was febrile with neck stiffness. Kernig's and Brudzinski signs of meningismus were positive. She had normal sensation and power in the lower extremities. Deep tendon reflexes in the lower extremities were normal. Also, perianal sensation and anal tone were normal. She did not have genital lesions and denied past history of sexually transmitted infections.

**Fig. 1:**
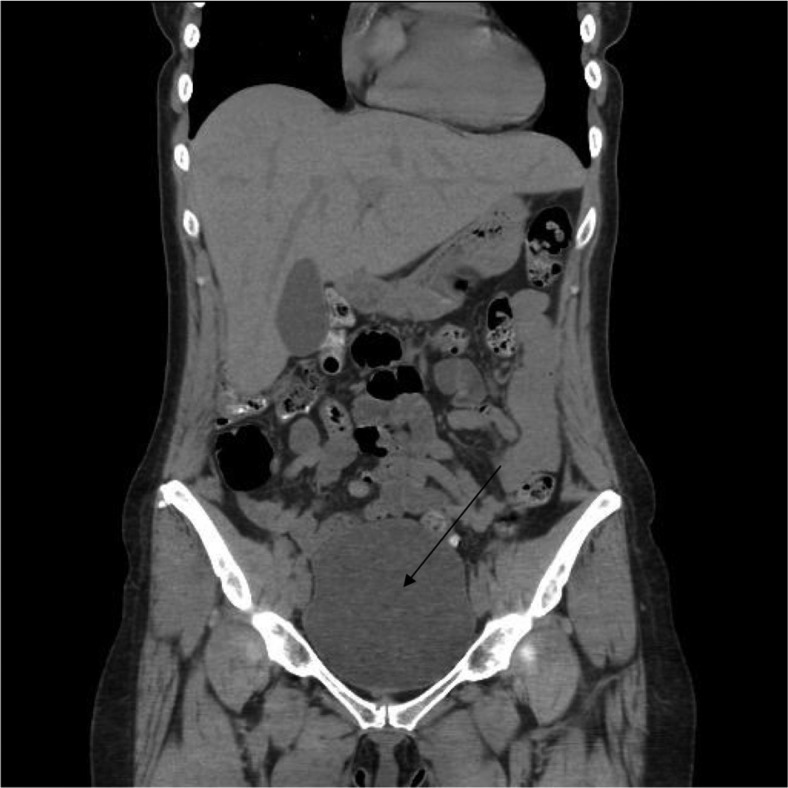
CT scan demonstrating distended urinary bladder in the pelvis.

**Fig. 2:**
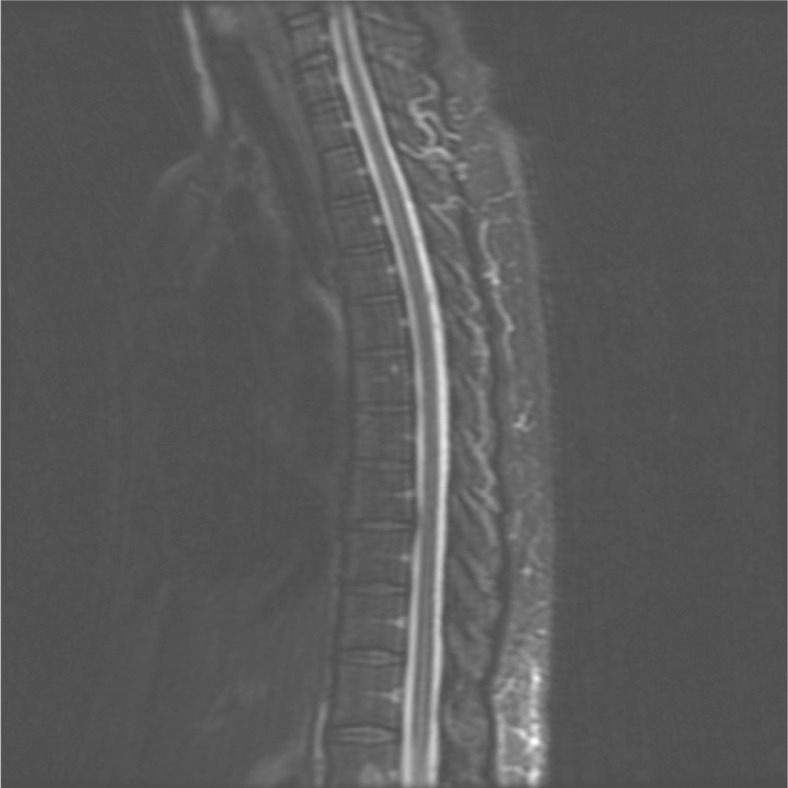
(A) MR scan, sag T2 Image, demonstrating no evidence of myelitis in this patient.

Bladder scan revealed post void urine of 750 ml, and a Foley catheter was placed. (Fig. 1). Unenhanced head CT was negative for any acute intracranial pathology (infarct, hemorrhage or mass). Brain and spine MRI did not reveal any features suggesting myelitis or radiculitis (Fig. 2). CSF analysis showed WBC of more than 700, predominantly lymphocytes, glucose of 50 mg/dl, protein 150 mg/dl and a negative gram stain. CSF was negative for Myelin basic protein and oligoclonal bands.

Due to predominance of lymphocytes in the CSF and a negative gram stain, empiric antiviral therapy was initiated with a 10 day course of intravenous Acyclovir at 10 mg/kg three times a day. Although her headache and photophobia subsided within 48 hours; she continued to have urinary retention and required intermittent urinary catheterization. Urodynamic study was undertaken on day 5 of presentation which confirmed the presence of atonic bladder.

She was then started on Bethanechol and Tamsulosin. At this point CSF-PCR came back confirming HSV-2 infection. CSF was negative for HSV1, Listeria, Cryptococcus, Tuberculosis, and VDRL.

She showed excellent recovery of bladder function within 2 weeks of onset of symptoms. A follow-up visit at 6 months confirmed persistence of her recovery.

## Discussion

According to the Centers for Disease Control and prevention (CDC) 2010, (www.cdc.gov/std/treatment/2001/STD), one in six Americans between the ages of 14–49 is infected with HSV-2. The infection rate is greater in women of up to one in three. Following initial infection, the virus establishes latency in the regional dermatomes with subsequent reactivation (3). Systemic complications of genital herpes include sacral radiculitis, meningitis, and non-genital distant skin lesions.

HSV-2 can be the causative organism in MRS and Elsberg syndrome, but the mechanism of urinary retention is different.

The neurological axis for bladder control is said to involve the cortex, splenium part of the corpus callosum, pons, spinal cord and its parasympathetic nerves to the bladder (4, 5).

Urinary retention in Elsberg syndrome is presumed to be due to reactivation of the Herpes virus in the sacral dorsal root ganglia with axonal spread to the spinal cord. This can usually be visualized as hyper intense T2 lesions on spinal MRI (2).

In MRS, the mechanism by which the meningitis causes urinary retention, without evidence of sacral radiculomyelitis is not clear. Detrusor hyporeflexia due unknown mechanism is regarded to be the cause of urinary retention (1).

Several hypotheses have been put forward to explain the detrusor hypofunction and urinary retention in MRS including spinal shock secondary to meningeal irritation, inflammation of upper motor neurons of the spinal cord, direct viral invasion, or development of post infectious acute disseminated encephalomyelitis (ADEM). Our patient had no symptoms or signs of encephalitis or myelitis and had normal CNS imaging.

A unique feature of our case is the early recovery of bladder function within 2 weeks contrary to the 4–8 weeks reported in the literature (1, 4). We hypothesize that early treatment with Acyclovir and bethanechol could have contributed to this good result. Acyclovir was started empirically on day-1 prior to the availability of the result of the HSV-2 PCR assay. The case also underscores unreliability of history in identifying patients with prior HSV-2 infections as many primary infections go unnoticed.

## Conclusion

Our case demonstrates the importance of recognizing MRS as a unique neuro-urological condition and a rare complication of a very common viral infection. The lack of history of sexually transmitted infections and lack of visible blisters, or signs and symptoms suggesting lumbosacral radiculomyelitis should not dissuade physicians from the pursuit of HSV-2 infection as the underlying cause of the urinary symptoms since early treatment with antiviral therapy may improve outcome.
